# Functional Enrichment, Drug Prediction, and Molecular Docking to Identify Fibroblast-Related Biomarkers for Gastric Cancer *via* High-Dimensional Weighted Gene Co-Expression Network Analysis

**DOI:** 10.2174/0118715303424481250929070902

**Published:** 2025-10-21

**Authors:** Hongpeng Lu, Suqi Lan, Xu Yuan, Peifei Li

**Affiliations:** 1 Department of Gastroenterology, The First Affiliated Hospital of Ningbo University, Ningbo, 315010, China;; 2 Department of Gastroenterology, Ninghai County Second Hospital, Ningbo, 315615, China

**Keywords:** Gastric cancer, cancer-associated fibroblast, high-dimensional WGCNA, drug prediction, epithelial-mesenchymal transition, immune escape

## Abstract

**Introduction:**

Cancer-associated fibroblasts (CAFs) can promote gastric cancer (GC) progression through regulating the tumor microenvironment (TME). This study explored cell-to-fibroblast communication based on the single-cell data of GC, identified CAF-related genes linked to GC using high-dimensional weighted gene co-expression network analysis (hdWGCNA), and conducted functional mining, drug prediction, and molecular docking for these genes.

**Materials and Methods:**

Single-cell data were preprocessed using the Seurat package. The communication network between cell subpopulations and fibroblasts was analyzed using CellChat. Key hub genes were initially identified through hdWGCNA, while differentially expressed genes (DEGs) between cancer and control groups were obtained using DESeq2. Subsequently, the overlapping genes between the hub genes and DEGs were subjected to LASSO regression (*via* the glmnet package) and SVM-RFE (implemented in e1071 package) to select biomarkers for GC. Immune cell infiltration was assessed using the CIBERSORT package, and functional enrichment analysis was performed on the background gene set by GSEA_4.2.2 software. Finally, drugs targeting the biomarkers were predicted by employing the DSigDB database.

**Results:**

Single-cell analysis identified eight major cell subpopulations, with fibroblasts distinctly marked by DCN and LUM expression. Cell communication analysis revealed that HLA-E-CD94:NKG2A and HLA-E-KLRC1 were the main interactions through which other cell clusters exerted influence on fibroblasts. *COL1A1* and *SERPINH1* were identified as CAF-related biomarkers that promoted GC progression through macrophage-mediated immune infiltration. High co-expression of the two genes was significantly enriched in epithelial-mesenchymal transition (EMT).

**Discussion:**

*COL1A1* and *SERPINH1* may promote GC progression *via* regulating EMT and forming an immunosuppressive microenvironment through ECM remodeling and macrophage polarization. Additionally, chitosamine was screened as a potential *COL1A1*-targeting drug for GC treatment.

**Conclusion:**

These findings have deepened our current understanding of CAF-mediated mechanisms in GC, contributing to the development of precision diagnostics and therapeutics in GC.

## 1 INTRODUCTION

Gastric cancer (GC) is a frequently detected malignant tumor [1, 2] that has a low 5-year survival rate and a high metastasis tendency [3]. *Helicobacter pylori* infection, as a leading cause of GC [4], exhibits a significantly higher incidence in male patients and in East Asia when compared to other regions [5]. As most GC cases are detected at advanced stages with poor prognosis, there is an urgent need to discover specific biomarkers for early diagnosis and precision therapy [6, 7]. Currently, systemic treatments for GC include targeted therapy, immunotherapy, and chemotherapy [8]. Although immune checkpoint blockade (ICB) therapy has demonstrated efficacy in the treatment of certain solid tumors, its response rate remains limited in advanced GC patients [9, 10].

As the most abundant stromal cell type in the Tumor Microenvironmen (TME), cancer-associated fibroblasts (CAFs) account for about 70% of the cellular composition of tumor tissues [11, 12]. CAFs have been shown to be involved in tumor invasion, drug resistance, and immunosuppressive microenvironment in GC by inducing chronic inflammation and secreting immunomodulatory cytokines [13]. Multiple studies have indicated that CAFs are closely associated with the treatment of GC, influencing the therapeutic outcomes of GC and holding potential as therapeutic targets [14, 15]. The single-cell RNA sequencing (scRNA-seq) technique can reveal the transcriptional heterogeneity of immune cells in TME [16, 17], providing a new approach to analyzing the cellular reprogramming and interaction networks in the TME of GC [18]. As a co-expression network analysis based on single-cell and spatial transcriptome data, High-Dimensional Weighted Gene Co-Expression Network Analysis (hdWGCNA) enables the study of gene co-expression patterns across multiple scales at cellular and spatial levels [19]. Studies found that compared to normal tissues, tumor-associated epithelial cells exhibit enhanced expression of oncogene signatures related to epithelial-mesenchymal transition (EMT) and cell motility, with the expression of GC oncogenes being particularly upregulated within the intestinal epithelial subpopulation [20]. However, current research remains largely confined to the characterization of single-cell atlases and lacks robust associations with biomarkers as well as exploration of potential therapeutic agents. Therefore, this study integrated single-cell sequencing to explore GC biomarkers related to CAFs and identify potential drugs for GC.

This study integrated single-cell transcriptomic data and multi-omics data from GC [21] and identified key gene modules related to CAFs using various computational methods to determine effective biomarkers for GC. The study systematically analyzed the association between biomarkers and immune infiltration and their enriched biological pathways, laying a scientific foundation for the identification of diagnostic biomarkers in GC. Additionally, virtual screening and molecular docking simulations using the DSigDB database and AutoDock Vina were performed to validate the interactions between the candidate biomarkers and targeted drugs. The current results provide potential intervention strategies to support the precision treatment of GC.

## 2 MATERIALS AND METHODS

### 2.1 Data Sources

This study included two datasets. The scRNA-seq data were obtained from Gene Expression Omnibus (GEO, https://www.ncbi.nlm.nih.gov/geo/download/?acc=GSE163558&format=file). Three tumor samples were obtained by downloading the single-cell dataset of GC (GSE163558). The transcriptome data were obtained from The Cancer Genome Atlas (TCGA) data, and the GC dataset was obtained from the Genomic Data Commons (GDC) database (https://portal.gdc.cancer.gov/) to retain the class A samples. Finally, 377 GC samples and 34 paracancerous control samples were collected.

### 2.2 Preprocessing of Single-cell Dataset

First, scRNA-seq data were read by the Seurat package [22], and the cells with the number of expressed genes between 200 and 10000 were filtered, resulting in 9446 cells. Then, the SCTransform method [22] was used to normalize the data, and the harmony package [23] was employed to eliminate batch effect among different samples after principal component analysis (PCA) downscaling. After UMAP dimensionality reduction, a K-Nearest Neighbors (KNN) plot was constructed using the FindNeighbors function [22], which calculated Euclidean distances based on the first 50 principal components (PCs). Finally, the FindCluster function [22] was used to cluster the cells at a resolution of 0.05. Cell types were identified based on the expression of known marker genes using the CellMarker2.0 database (http://117.50.127.228/ CellMarker/) [24].

### 2.3 Screening of Single-cell Subpopulation-Specific Genes

FindAllMarkers [22] was used to screen genes that are highly expressed across different subpopulations (log_2_ fold change (logfc) threshold = 0.25, min.pct = 0.30, only.pos = T).

### 2.4 Cell Communication Analysis

Using CellChat [25], we identified ligand-receptor pairs through which other cell subpopulations acted on fibroblasts by selecting “Cell-Cell Contact” as the interaction type.

### 2.5 Using hdWGCNA to Screen CAFs

The R data serialized (.rds) files obtained from single-cell annotation were imported into hdWGCNA [19] to develop a co-expression network based on CAFs. The KNN algorithm (k=25) was then applied to aggregate cells into metacells, effectively reducing the sparsity of the single-cell expression matrix. The maximum shared cell number between any two metacells was set to 1. Subsequently, an optimal soft-thresholding power was calculated to establish a scale-free co-expression network, from which modules of highly correlated genes were identified. Then, the key hub genes in the modules were determined based on the connectivity of the nodes within the modules.

### 2.6 Screening Differentially Expressed Genes (DEGs)

The samples from the GEO dataset were divided into disease and control groups, and the DESeq2 package [26] was used to compare the gene expression differences between the two groups. DEGs were screened under the criteria of |log_2_ fold change (FC)| ≥ 1 and *p* < 0.01. The key hub genes and DEGs were intersected to identify common genes.

The locations of the intersected genes on the chromosomes were retrieved from the Ensembl database (https://www.ensembl.org/index.html), and their distribution was visualized using the Circos database (http://circos.ca/).

### 2.7 Screening Biomarkers by Machine Learning Algorithms

The upregulated intersected genes were subjected to LASSO regression analysis using the glmnet package [27], and the model with the smallest error was taken as the result of LASSO analysis. SVM-RFE analysis was performed on the upregulated common genes with the e1071 package [28], and the model with the least error was selected. The common genes screened by both machine learning models were considered biomarkers for GC.

### 2.8 Immune Infiltration Analysis

The CIBERSORT package in R [29] was used to calculate the infiltration of immune cells in the samples of the TCGA dataset, and the leukocyte signature matrix (LM22) was obtained from the official CIBERSORT website (https://cibersortx.stanford.edu/).

### 2.9 Gene Set Enrichment Analysis

The samples were divided by the median expression of biomarkers. Utilizing GSEA_4.2.2 software (FDR < 0.05), enrichment analysis was performed on the background gene set collected from the HALLMARK gene set.

### 2.10 Targeted Drug Prediction and Molecular Docking

The biomarkers were imported into the Enrichr package [30] for GSEA. DSigDB (https://dsigdb.tanlab.org/DSigDBv2.0/) was employed to predict the biomarker-targeted drugs, and then the crystal structures of the receptor proteins were obtained from the UniProt database (https://www.uniprot.org/). The screening began with the selection of the X-ray structures. These structures were then sorted by resolution (unit: Ångström (Å)), where a smaller value indicates higher resolution and accuracy of the structure, and subsequently by crystal structures, where longer coverage provides more complete structural information (*e.g.*, binding sites). Structures with higher resolution and longer coverage were prioritized. Based on the drug prediction results, the 3D structures of the targeted drugs were downloaded from the PubChem website (https://pubchem.ncbi.nlm.nih.gov/) as ligands. When a drug lacked a 3D structure or required overexpression or knockdown of a gene, other drugs were selected according to the P value, from the lowest to the highest, in a sequential order [31].

All molecules were pre-treated by PyMOL software [32] to remove water molecules, hydrogenate, and eliminate small molecule ligands. The 3D structures of the target drugs were then energy-minimized using ChemBioOffice [33] to obtain stable conformations. Subsequently, molecular docking simulations between the receptor proteins and drug molecules were performed by employing AutoDockTools [34]. The docking results were screened according to the binding energy parameter (screening threshold set at < -5 kcal/mol), and ligand-receptor combinations with strong binding affinity were retained.

### 2.11 Statistical Analysis

All statistical analyses were conducted using the R language (version 3.6.0). The Wilcoxon rank-sum test was carried out to assess the statistical significance of differences in immune infiltration between the tumor and control groups. The associations between the biomarkers and immune cell infiltration were evaluated using Spearman's correlation coefficient. A *p* < 0.05 was regarded as a statistically significant difference, and ns was considered as not significant (*p* > 0.05).

## 3 RESULTS

### 3.1 Single-cell Mapping for GC

**Fig. (1):**
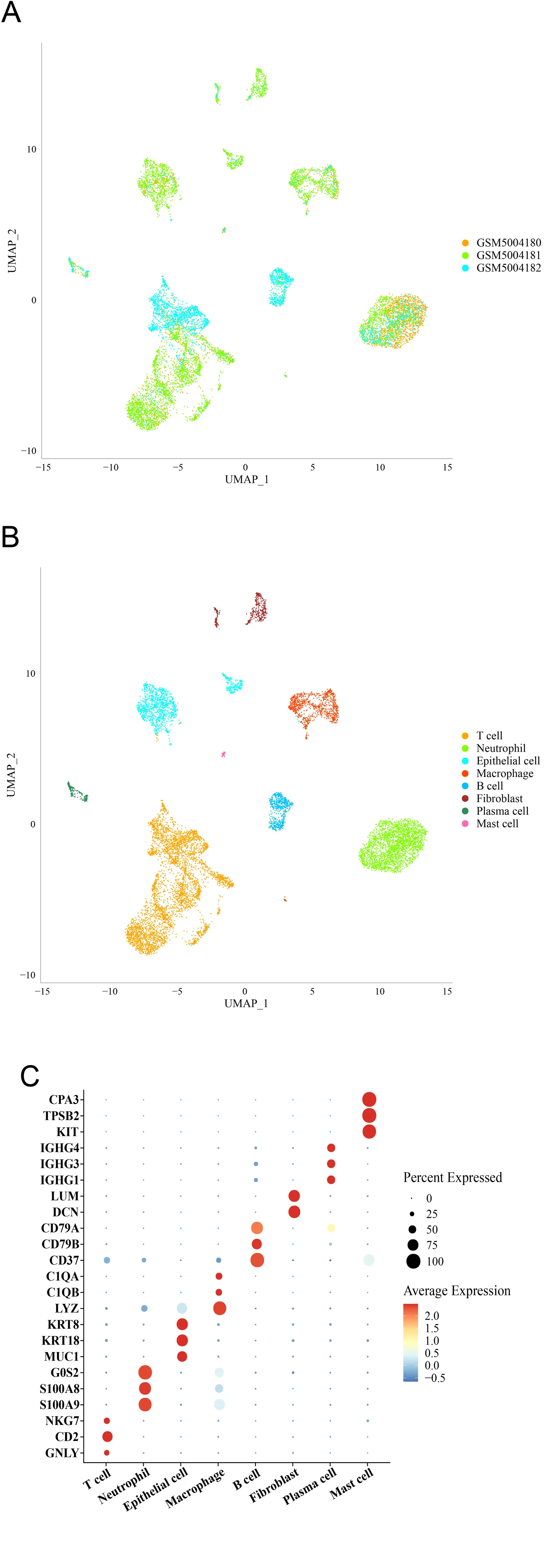
Single-cell dataset mapping analysis of GC. (**A**) Distribution of different samples after batch correction. (**B**) Distribution of different cell types visualized by UMAP. (**C**) The expression levels of marker genes in different cells.

After preprocessing the single-cell dataset, we identified eight major cellular subpopulations (Figs. **1A** and **B**). Using the CellMarker2.0 database, genes specific to the eight single-cell subpopulations were identified based on their relevant markers. T cells were identified by the genes *GNLY*, *CD2*, and *NKG7*; neutrophils were identified by *S100A9*, *S100A8*, and *G0S2*; epithelial cells were identified by *MUC1*, *KRT18*, and *KRT8*; macrophages were identified by *LYZ*, *C1QB*, and *C1QA*; B cells were identified by *CD37*, *CD79B*, and *CD79A*; fibroblasts were identified by *DCN* and *LUM*; plasma cells were identified by *IGHG1*, *IGHG3*, and *IGHG4*; and mast cells were identified by *KIT*, *TPSB2*, and *CPA3* (Fig. **1C**).

### 3.2 Cell Communication Analysis

**Fig. (2):**
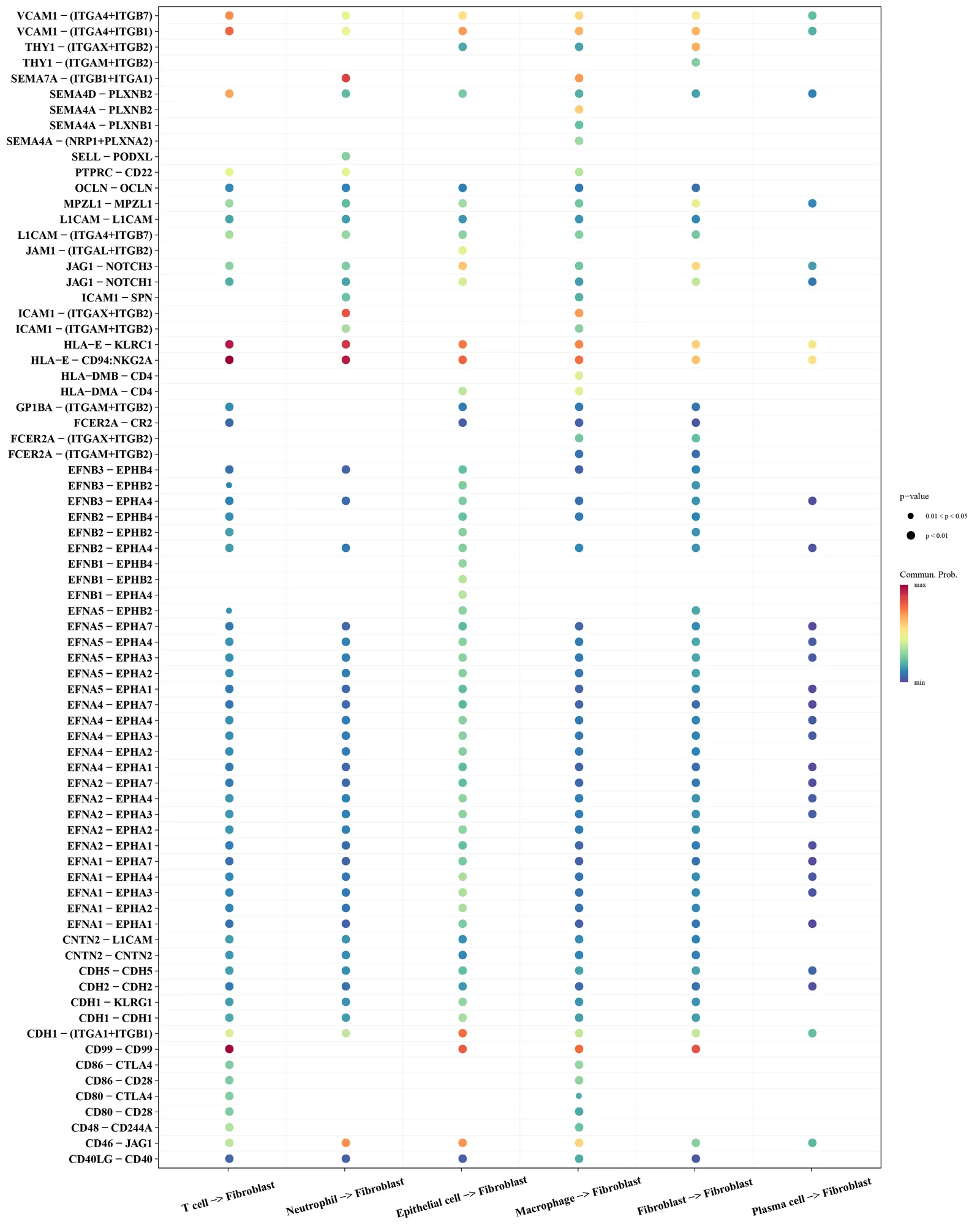
Receptor-ligand bubble diagram of all cells regulating GC.

We further investigated the ligand-receptor pairs through which other cell subpopulations communicate with fibroblasts and extracted the ligand-receptor information for each cluster. The results showed that all six clusters, namely, T cells, neutrophils, epithelial cells, macrophages, fibroblasts, and plasma cells, affected fibroblasts through cell communications of HLA-E-CD94:NKG2A and HLA-E-KLRC1 (Fig. **2**).

### 3.3 CAF-related Modules Identified by hdWGCNA

**Fig. (3):**
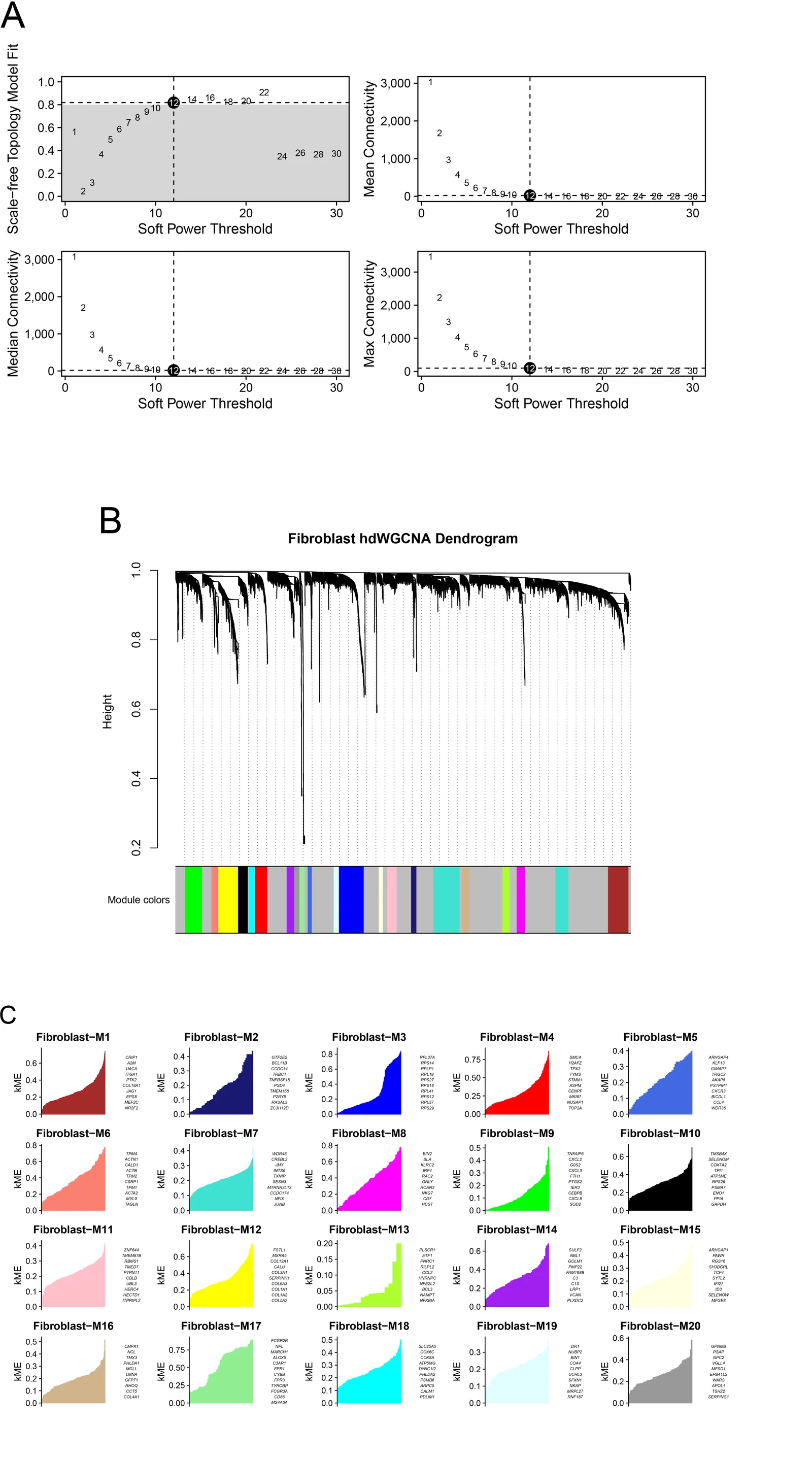
Acquisition of CAF-related modules. (**A**) Soft threshold screening to obtain the optimal soft threshold of 12. (**B**) Co-expression network tree, the upper half is the gene tree, each branch represents a gene, and the lower half is the module corresponding to the gene. (**C**) Module partitioning, the vertical coordinate is the correlation to kME value, which represents the connectivity of each gene based on the feature genes, and the right side is the module's hub genes.

The optimal soft threshold of 12 was determined by hdWGCNA calculation (Fig. **3A**) to develop a co-expression network (Fig. **3B**). Subsequently, we calculated the module eigengene (ME) to represent the expression profile of each module. Hub genes within each module were then screened based on their module connectivity, resulting in a total of 20 modules (Fig. **3C**).

**Fig. (4):**
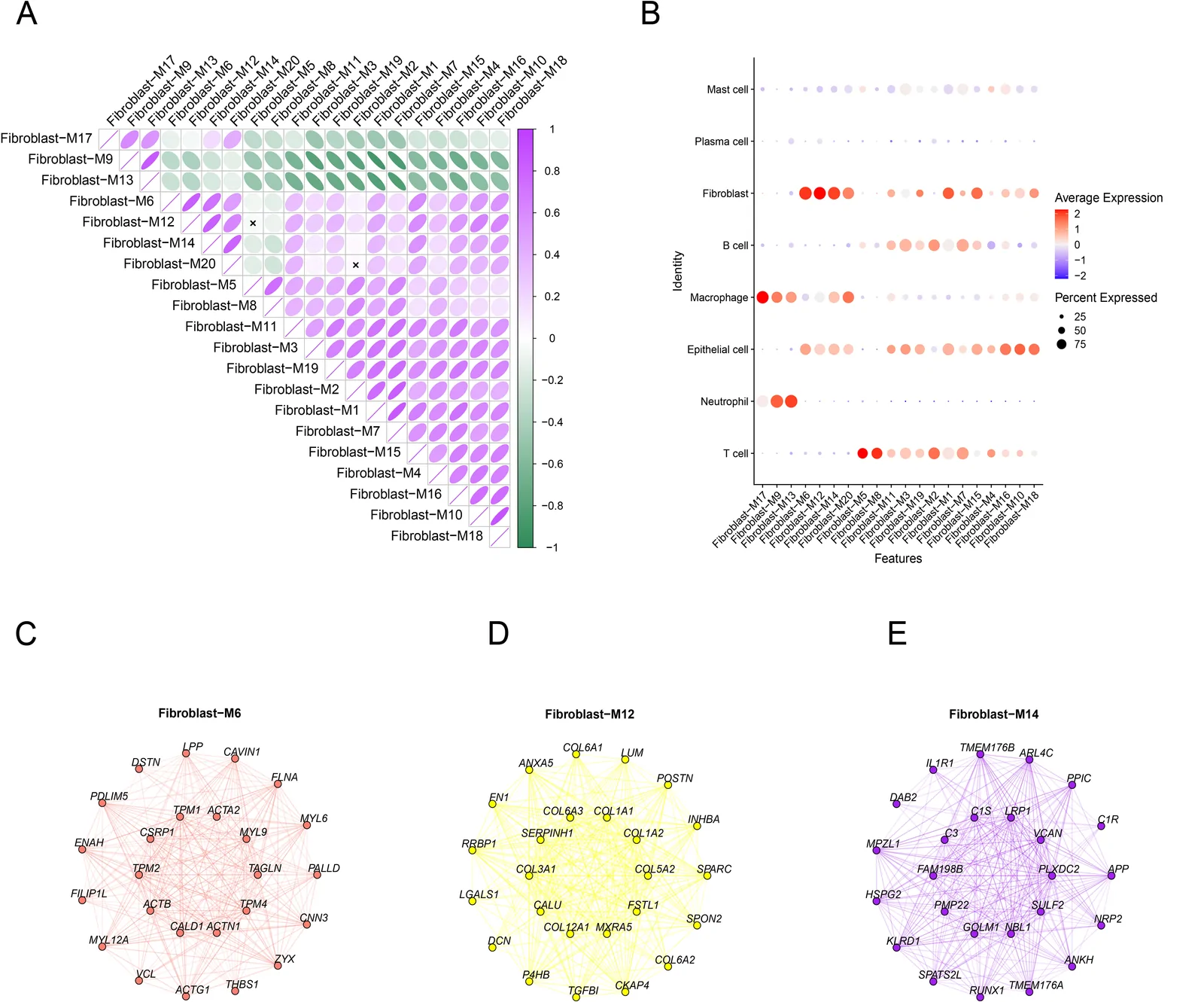
Acquisition of hub genes. (**A**) Module correlation matrix. Where, purple color represents positive correlation and green color represents negative correlation, and the correlation transformation follows the colorbar transformation on the right side. (**B**) Expression of genes within the module in different cells, where red represents high expression, blue represents low expression, and the size of the circle represents the proportion of cells. (**C-E**) Co-expression network of hub genes within the modules M6, M12, and M14. The inner circle represents the key genes, and the outer circle represents the secondary key genes.

The expression of genes within each module across different cell types was calculated, and an inter-module correlation matrix was plotted (Fig. **4A**). The results showed that the genes within the modules M6, M12, and M14 had higher expression, especially in fibroblasts (Fig. **4B**). Therefore, these three modules were selected as the key modules, and the hub gene network was developed (Figs. **4C**-**E**). Specifically, 10 genes in the inner circle were identified as key genes, while 15 genes in the outer circle were designated as secondary key genes.

### 3.4 Differential Expression Analysis

**Fig. (5):**
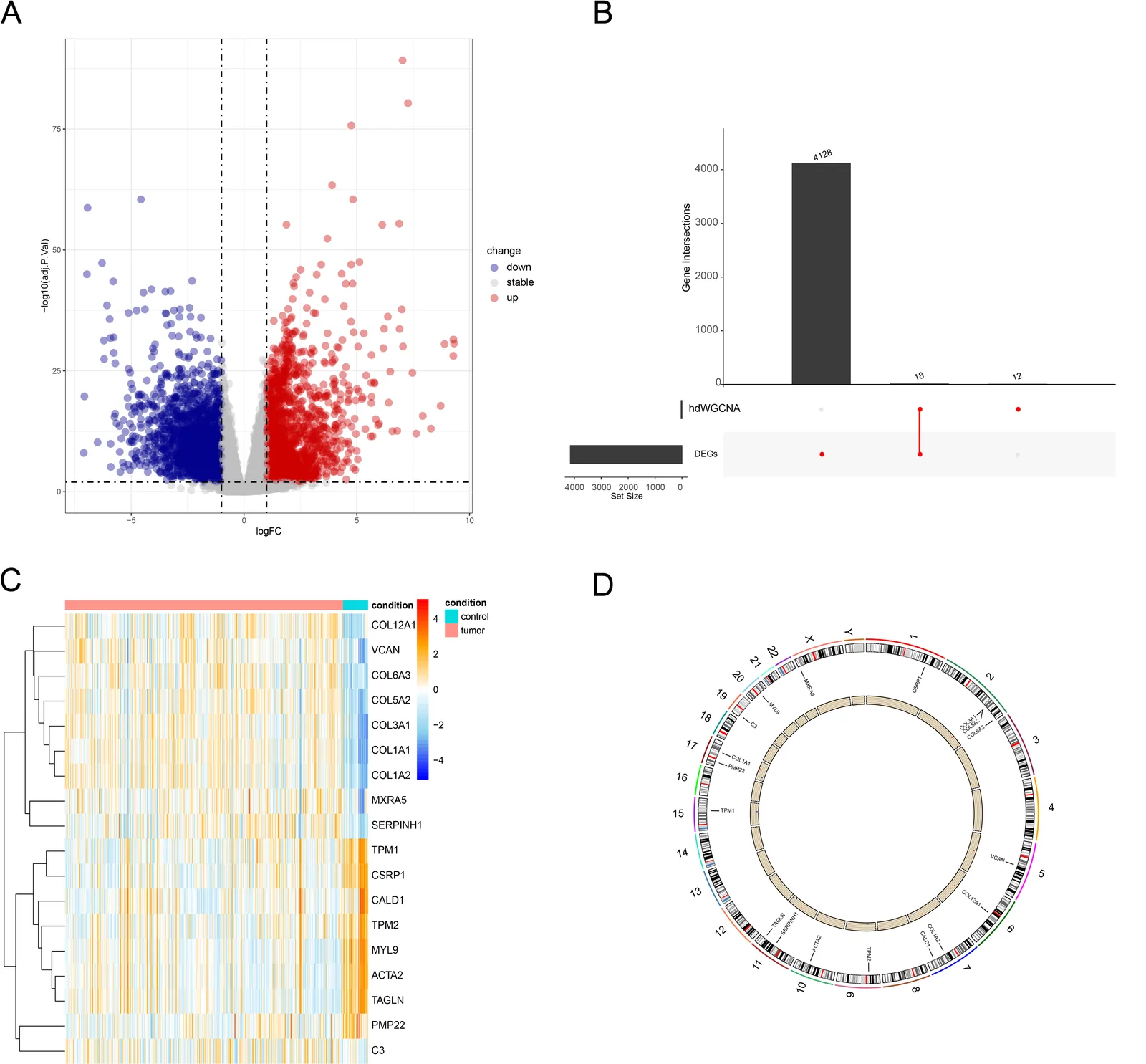
Acquisition of DEGs. (**A**) Volcano plot of DEGs distribution. Red represents differentially up-regulated genes, blue represents differentially down-regulated genes, and gray represents non-differentially expressed genes. (**B**) Upset plots of DEGs as well as the hdWGCNA module hub genes. The left bar shows the number of each subset of genes, and the top bar shows the number of each intersected gene. (**C**) Heatmap showing the expression of the 18 intersected genes. Blue represents low expression, and red represents high expression. (**D**) Chromosome distribution of the 18 intersected genes. The outermost circle in the graph represents the chromosome, the middle circle represents the position of the gene on the chromosome, and the inner circle represents the differential expression of the gene.

A total of 4146 DEGs, including 2183 downregulated genes and 1963 upregulated genes, were acquired (Fig. **5A**). The hub genes selected by hdWGCNA were intersected with DEGs, resulting in 18 common genes (10 upregulated genes and 8 downregulated genes) (Fig. **5B**). The expression heatmap of the 18 intersected genes was drawn (Fig. **5C**), and their locations on the chromosomes were visualized (Fig. **5D**).

### 3.5 Two Machine Learning Algorithms to Screen GC Biomarkers

**Fig. (6):**
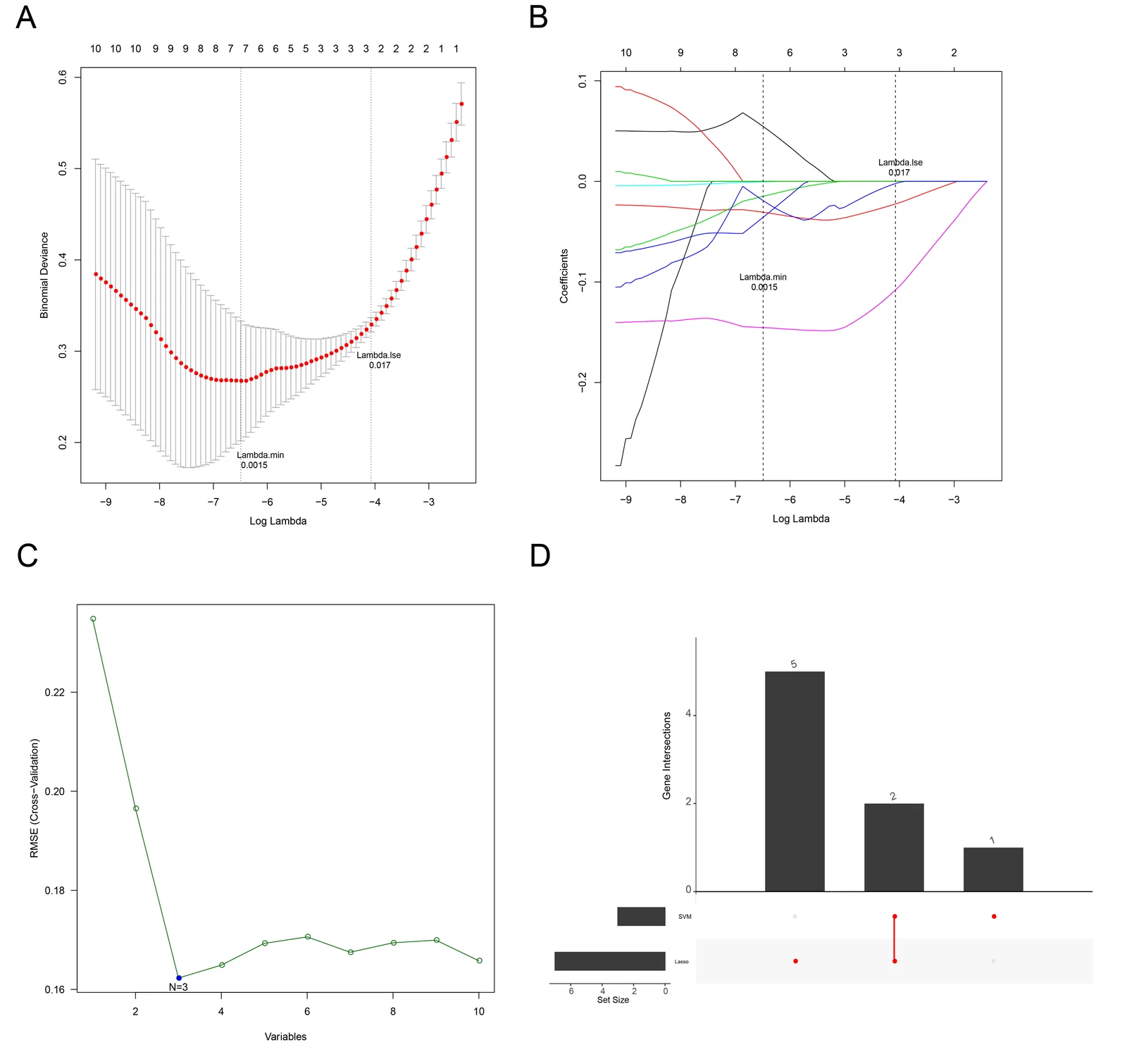
Acquisition of biomarkers. (**A**) Plot of LASSO penalty term parameters. Horizontal coordinates represent log(lambda) values, and vertical coordinates represent degrees of freedom, indicating cross-validation errors. (**B**) LASSO regression coefficients plot. Horizontal coordinates are log(lambda) and vertical coordinates are the coefficients of the genes, showing how the coefficients of the different variables change with the λ-penalty. (**C**) Plot of SVM-RFE generalized error versus the number of features. The horizontal coordinate represents the number of genes included within the model at each iteration, and the vertical coordinate represents the root mean square error, with the smallest value indicating the optimal model. (**D**) Intersection analysis plot of LASSO and SVM-RFE for upregulated intersected genes.

The ten upregulated intersecting genes were further refined using machine learning. In LASSO regression analysis, the model yielded the least error (λ.min = 0.0015) with seven feature genes (Figs. **6A** and **6B**). SVM-RFE analysis showed that the model had the least error (N=3) with three feature genes (Fig. **6C**). *COL1A1* and *SERPINH1* were the common genes in the intersection of the results from the two algorithms and were therefore considered biomarkers for GC (Fig. **6D**).

### 3.6 Correlation between Immune Infiltration and the Biomarkers

**Fig. (7):**
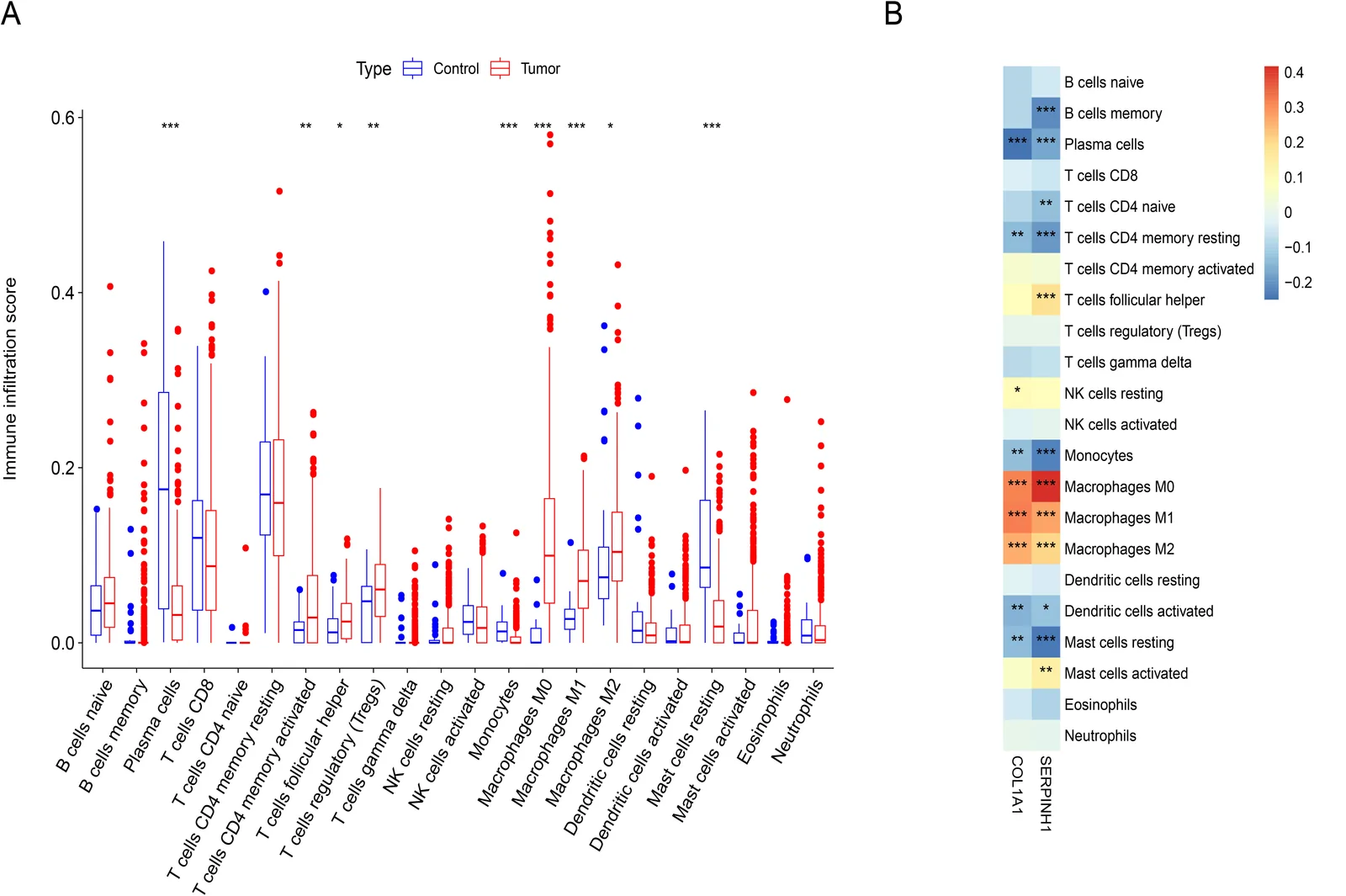
Correlation of biomarkers with immune infiltration. (**A**) Box line plot of immune cell infiltration in tumor and control groups. (**B**) Spearman correlation analysis of *COL1A1*, *SERPINH1,* and immune infiltration. And * means *p* < 0.05, ** means *p* < 0.01, *** means *p* < 0.001.

The results of immune infiltration analysis showed that the infiltration score of T cells CD4 memory activated, T cells follicular helper, Tregs, macrophages M0, macrophages M1, and macrophages M2 was significantly higher in the tumor group than in the control group (Fig. **7A**, *p* < 0.05), while the expression of plasma cells, monocytes, and mast cells resting was markedly higher in the control group than in the tumor group (Fig. **7A**, *p* < 0.05). Calculation of the correlation coefficients between the expression of the two genes and immune cell infiltration scores revealed that the biomarkers were correlated with the infiltration of most immune cell types, particularly with macrophages (M0, M1, and M2) (Fig. **7B**, *p* < 0.05).

### 3.7 Gene Set Enrichment Analysis (GSEA)

**Fig. (8):**
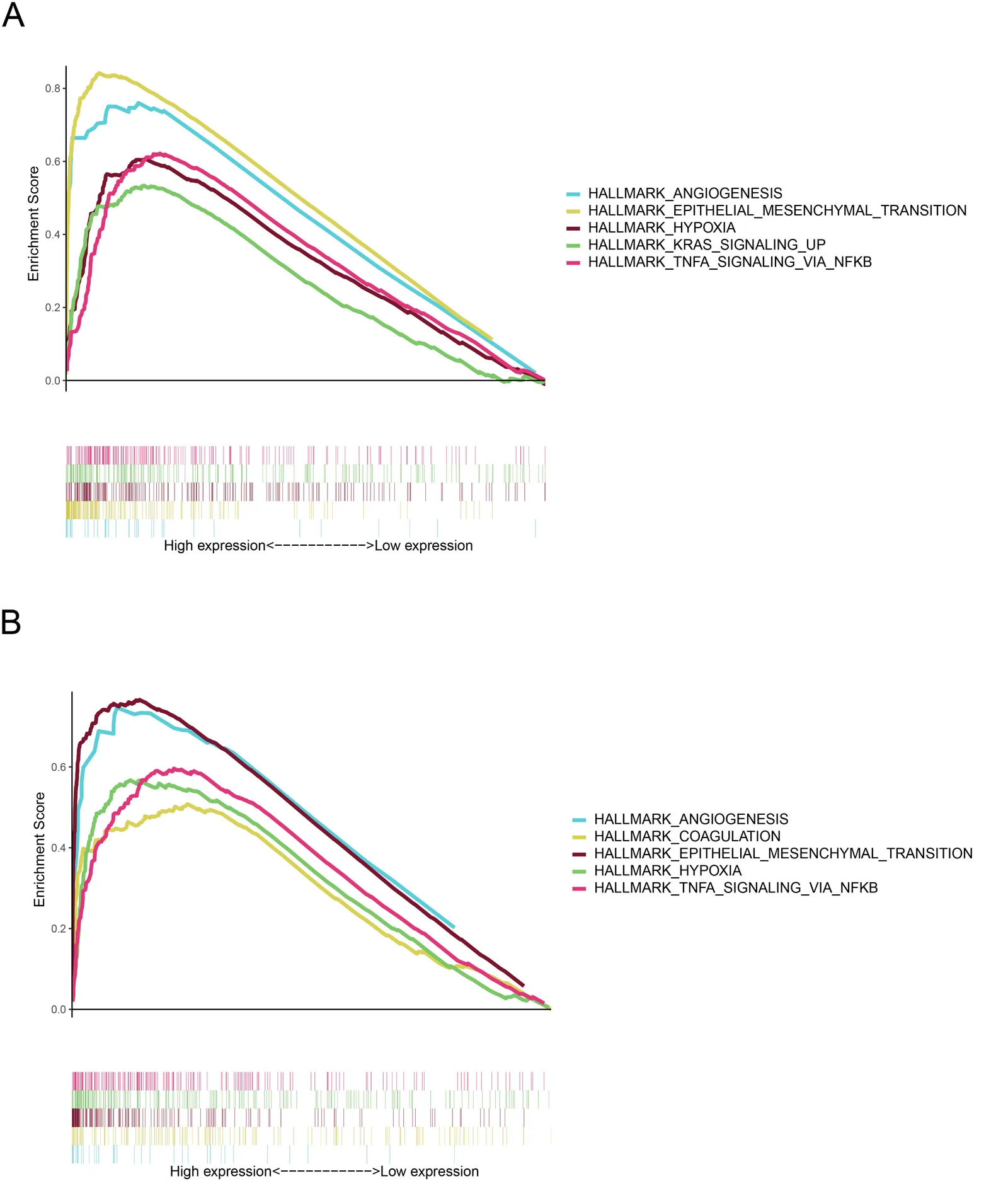
Results of enrichment analysis. (**A**) Results of GSEA enrichment analysis for high and low *COL1A1* expression groupings. The upper panel illustrates the dynamic calculation trajectory of the Enrichment Score (ES), which is based on genes ranked by their correlation or differential expression with *COL1A1*. The ES is incremented when genes within the predefined set are encountered and decremented when outside genes are met, generating a curve where the peak or trough represents the gene set's final ES value. A positive ES indicates significant enrichment at the top of the ranked list, associating with high *COL1A1* expression, whereas a negative ES suggests enrichment at the bottom, linking to low *COL1A1* expression. The lower panel shows gene expression distributions within this pathway, with a heatmap depicting differential expression patterns between *COL1A1*-high and -low groups. (**B**) Results of GSEA enrichment analysis of *SERPINH1* expression in high and low groups.

GSEA revealed that gene sets related to hypoxia response, EMT, angiogenesis, and TNF-α signaling *via* NF-κB were significantly enriched in samples with high co-expression of *COL1A1* and *SERPINH1* compared to low-expression groups (Figs. **8A** and **B**).

### 3.8 Drug Prediction and Molecular Docking

**Fig. (9):**
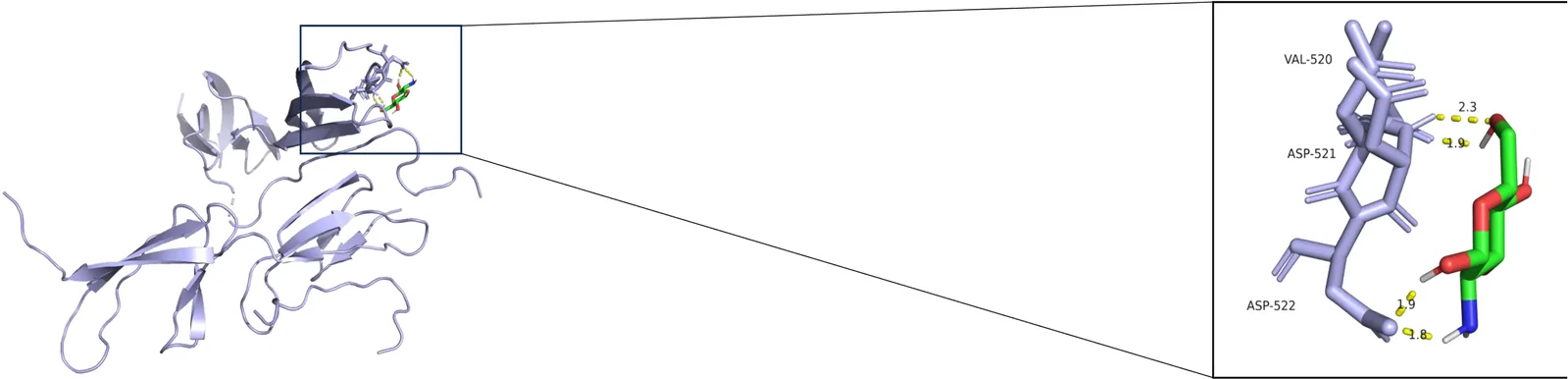
Molecular docking results. In the figure, blue molecules represent receptor proteins, green molecules represent small drug molecules, and cyan represents amino acids. The binding hydrogen bond between the receptor protein and the drug small molecule is indicated by the yellow dotted line, and the number above is the hydrogen bond length (Å).

Using the DSigDB database, biomarker-targeted drug prediction was performed. After screening, chitosamine was identified as a potential drug for GC. The crystal structure of *COL1A1* (3ejh) was found for the protein, while no valid crystal structure was found for *SERPINH1*. The docking results revealed that the binding energy of both molecules was -5.8 kcal/mol and that the hydrogen bond lengths were within the normal range (Fig. **9**).

## 4 DISCUSSION

GC is a major public health concern worldwide [35]. CAFs play critical pro-carcinogenic roles in the TME [36], and their interactions with GC cells could activate signaling pathways related to cell adhesion and metastasis, thereby promoting tumor progression [37]. Given the crucial role of CAFs in GC, this study employed computational technologies, such as hdWGCNA, to screen two CAF-related biomarkers (*COL1A1* and *SERPINH1*) for GC. Through virtual screening and molecular docking simulations, chitosamine was identified as a potential targeted drug for *COL1A1*. These findings provide potential diagnostic markers and therapeutic targets for the precision treatment of GC.

### 4.1 The Development of GC and Cell Communication Analysis

This study found that HLA-E-CD94:NKG2A and HLA-E-KLRC1 were the major forms of cellular communication between other cell clusters and fibroblasts in the TME of GC. Human leukocyte antigen E (HLA-E), a non-classical class Ib major histocompatibility complex (MHC) [38], is recognized by CD94/NKG2A receptors on the surface of NK cells and T cell subsets by presenting conserved sequences of MHC-I signal peptides [39]. In GC, HLA-E was significantly enriched in tumor-infiltrating macrophages, and tumor-infiltrating lymphocytes (TILs) have co-enrichment of programmed cell death protein 1 (PD-1) and CD94/NKG2A. Tumor cells evade immune attack by upregulating HLA-E expression and presenting tumor-associated peptides, which persistently activate the CD94/NKG2A inhibitory pathway and inhibit the normal function of NK cells and cytotoxic/depleted CD8^+^ T cells [40, 41]. *KLRC1* encodes the NKG2A protein, and its encoded product mediates the above inhibitory effects through binding to HLA-E [42]. Anti-*KLRC1* (NKG2A) monoclonal antibodies and *KLRC1* knockout can significantly enhance the cytotoxic activity of NK cells against HLA-E^+^ tumors through deregulation [43]. This indicated that the HLA-E-CD94:NKG2A and HLA-E-KLRC1 signaling axes may influence GC development through tumor immune escape.

### 4.2 The Functional Significance of *COL1A1* and *SERPINH1*

With the application of computational technology, the research of GC-related prognostic models has been continuously deepened [44]. Although previous studies have identified CAF-related biomarkers in GC [45, 46] and constructed prognostic models incorporating *COL1A1* and *SERPINH1* [47], most of them did not examine the interactions between these two biomarkers. This study used hdWGCNA to classify spatial co-expression network modules based on single-cell datasets and combined with machine learning methods to screen *COL1A1* and *SERPINH1* as the biomarkers [48] for constructing a prognostic model for GC [49]. *COL1A1*, a gene encoding collagen type I alpha 1 chain and a well-established marker for CAFs, is predominantly localized to the extracellular matrix (ECM) [50]. Functionally, *COL1A1* contributes to the process of EMT, while silencing *COL1A1* can suppress the proliferation, migration, and invasion of GC cells [51]. It was reported that *COL1A1* enhances GC cell invasion and proliferation through the Phosphatidylinositol 3-kinase/ Protein Kinase B (PI3K/AKT) signaling pathway [52] or the Angiopoietin-like Protein 4/Syndecan-4 (ANGPTL4/SDC4) axis [53]. *SERPINH1*, also known as serpin protease inhibitor clade H1, encodes heat shock protein 47 (HSP47), which is involved in the proper folding and secretion of various collagens [51]. Notably, the aberrant expression of *SERPINH1* has been detected in the clinical specimens of GC [54]. *SERPINH1* can also regulate EMT through modulating the Wnt/β-catenin signaling pathway, which in turn affects GC development [55]. Additionally, enrichment analysis showed that *COL1A1* and *SERPINH1* were significantly enriched in EMT. These findings suggested that *COL1A1* and *SERPINH1* may affect GC invasion and metastasis by synergistically regulating EMT, but the specific mechanisms required further exploration. In addition to the ECM pathway, high-expressed *COL1A1* and *SERPINH1* were also associated with the TNF-α signaling *via* NF-κB pathway. It was found that *H. pylori*-induced NF-κB-PIEZO1-YAP1-CTGF axis drives GC progression and CAF-mediated TME remodeling [56]. Moreover, activation of the TNF-α/NF-κB signaling pathway promotes the infiltration and functional activation of immune cells (*e.g.*, NK cells, CD8^+^ T cells) but suppresses the immunosuppressive effects of stromal cells, such as fibroblasts [57].

### 4.3 The Role of CAFs in GC

CAFs are the most abundant cell type in the TME of GC. Activated CAFs form an extensive network of signaling interactions with immune cells through the deposition and remodeling of the ECM to mediate GC progression [58]. Our immune infiltration analysis revealed that *COL1A1* and *SERPINH1* primarily affected GC development through macrophages, with the expression of macrophages in tumor tissues being significantly higher than in controls. CAF-macrophage crosstalk has been shown to modulate the response of peritoneal metastases of GC to ICB [59]. In addition, CAFs interact with macrophages in the TME by secreting extracellular vesicles (EVs), which promote macrophage polarization from pro-inflammatory M1 to pro-tumor M2 phenotype. This process is often mediated by EVs carrying pro-tumorigenic molecules to drive GC cell proliferation [60]. Another study found that *IGFBP7* secreted by CAFs enhances tumor-associated macrophage (TAM) polarization *via* the FGF2/FGFR1/PI3K/AKT axis [61]. M2-like polarized macrophages play a role in promoting tumorigenesis and evading immune surveillance, thereby facilitating GC progression [62]. Taken together, this study hypothesized that *COL1A1* and *SERPINH1* promote the development of GC by influencing macrophage polarization, specifically inducing the polarization of M1 macrophages to M2 phenotype, which, in turn, facilitates tumorigenesis and immune surveillance evasion in GC.

### 4.4 The Therapeutic Potential of Chitosamine

In this study, we predicted targeted drugs for *COL1A1* and *SERPINH1,* providing a range of candidate drugs for molecular docking validation. The docking simulations evaluated the binding affinity between these drugs and the target proteins, thereby verifying the prediction rationale from a molecular interaction perspective. This integrated approach ultimately identified chitosamine (*i.e*., glucosamine) as a potential drug targeting *COL1A1*. Glucosamine, an aminosugar naturally present in cartilage and synovial fluid, is an amino derivative of glucose used to relieve joint pain in clinical practice. Glucosamine supplementation improves intestinal inflammatory symptoms in the models of inflammatory bowel disease (IBD). Glucosamine has also been shown to help relieve bloating and regulate intestinal microecology in gastrointestinal diseases [63]. It has been reported that glucosamine secreted by CAF affects cancer development by upregulating *HSD3B1* [64], but its specific role in GC requires further experimental verification. However, the identification of chitosamine as a potential drug targeting *COL1A1* was solely based on computational analysis and molecular docking simulations, which require further experimental validation.

### 4.5 Limitations

This study also has limitations. Firstly, the findings that *COL1A1* and *SERPINH1* may synergistically regulate EMT and tumor progression were obtained solely based on computational analysis and lacked experimental validation. Thus, the molecular mechanism of *COL1A1*/*SERPINH1* in regulating fibroblast activation should be validated through *in vitro* experiments, including co-culture of CAFs with GC cells and macrophages, as well as *in vivo* xenograft models in nude mice. Secondly, this study had a relatively small sample size and relied on public datasets with limited clinical annotation. Additionally, immune infiltration analysis was performed using bulk RNA-seq data rather than single-cell resolution data, which may compromise the granularity of the analysis to some extent. Our future analysis will expand the sample size to include clinical samples from multiple centers and different geographical regions, thereby covering a wider range of GC subtypes. Finally, cellular communication patterns and enrichment pathways were identified based on computational analysis, which also requires further verification by cellular experiments, animal models, and other experiments.

## CONCLUSION

In this study, *COL1A1* and *SERPINH1* were identified as two CAF-related biomarkers for GC, providing new potential molecular targets for early prediction and treatment of GC. Immune infiltration and enrichment analysis revealed that *COL1A1* and *SERPINH1* may affect GC development through ECM remodeling. Drug screening and molecular docking identified chitosamine as a potential target for COL1A1, and its binding mode with the *COL1A1* protein was simulated. These findings could deepen our understanding of the mechanistic roles of CAFs in GC and establish a “biomarker-pathway-targeted drug” research pipeline for clinical translation in the precision diagnostics and treatment for GC.

## Data Availability

The datasets generated and/or analyzed during the current study are available in the (GSE163558) repository (https://www.ncbi.nlm.nih.gov/geo/query/acc.cgi?acc= GSE163558).

## LIST OF ABBREVIATIONS

GC: Gastric Cancer
H. pylori: Helicobacter pylori
ICB: Immune Checkpoint Blockade
TME: Tumor Microenvironment
CAF: Cancer-Associated Fibroblast
scRNA-seq: Single-cell RNA sequencing
hdWGCNA: High-Dimensional Weighted Gene Co-Expression Network Analysis
GEO: Gene Expression Omnibus
TCGA: The Cancer Genome Atlas
GDC: Genomic Data Commons
PCA: Principal Component Analysis
UMAP: Uniform Manifold Approximation And Projection
KNN: K-Nearest Neighbors
RDS: R data serialized
DEG: Differentially Expressed Gene
FC: Fold Change
LASSO: Least Absolute Shrinkage and Selection Operator
SVM-RFE: Support Vector Machine-Recursive Feature Elimination
LM22: Leukocyte Signature Matrix
Å: Ångström
ME: Module Eigengene
Tregs: Regulatory T cells
EMT: Epithelial-Mesenchymal Transition
GSEA: Gene Set Enrichment Analysis
TNF-α: Tumor Necrosis Factor α
NF-κB: Nuclear Factor κB
HLA-E: Human Leukocyte Antigen E
MHC: Major Histocompatibility Complex
NK: Natural Killer
TIL: Tumor-Infiltrating Lymphocyte
PD-1: Programmed Cell Death Protein 1
COL1A1: Collagen Type I Alpha 1
ECM: Extracellular Matrix
SERPINH1: Serpin Protease Inhibitor Clade H1
PI3K/AKT: Phosphatidylinositol 3-kinase/Protein Kinase B
ANGPTL4/SDC4: Angiopoietin-like Protein 4/Syndecan-4
HSP47: Heat Shock Protein 47
EV: Extracellular Vesicle
IBD: Inflammatory Bowel Disease
TAM: Tumor-Associated Macrophage
ES: Enrichment Score
kME: Correlation to ME
